# Complete chloroplast genome sequence of *Solanum erianthum*: genome structure and genomic resources

**DOI:** 10.1080/23802359.2021.1981162

**Published:** 2021-09-27

**Authors:** Dan Li, Zhan-Lin Liu

**Affiliations:** Life Science College, Northwest University, Xi’an, China

**Keywords:** Chloroplast genome, *Solanum erianthum*, genome structure

## Abstract

*Solanum erianthum* is known for its valuable medicinal properties. In this study, we report its complete chloroplast genome. The chloroplast genome size is 156,343 bp, including a LSC region of 86,855 bp, a SSC region of 18,608 bp and two inverted repeats (IR). The complete chloroplast genome includes a total of 128 unique genes with 83 protein-coding sequences, 37 tRNA and 8 rRNA genes. The results showed that *S. erianthum* was the most closely related to *S. violaceum*.

*Solanum erianthum* is belonged to the genus *Solanum* (Solanaceae), which is widespread in tropical Asia, Oceania and South America. And it’s because of its wide distribution, *S. erianthusm* have ten synonym such as *S. adulterinum* Buch.-Ham. ex Wall., *S. amblycalyx* Dunal, *S. eriocalyx* Dunal and so on (POWO 2021). In China, it mainly distributed in Fujian, Guangdong, Guangxi, Guizhou, Hainan, Sichuan, Taiwan, Xizang and Yunnan provinces of China (Zhang et al. 1994). It’s a kind of ethnic medicinal plant in China, also in India, Mexico, Spain and other countries, with main treatment is stomachache, abdominal pain, ventilation, etc. (You et al. 2018) . Genomics and modern biotechnology can be used to study the molecular mechanisms of medicinal ingredients. In this study, we sequenced and analyzed the chloroplast genome of *S. erianthum*, aiming to provide its chloroplast genome structure and valuable genomic resources.

The samples is collected from Zhaotong city, Yunnan Province of China (28°15'10.24″N, 103°47'1.30″E), and the specimens are deposited in the Herbarium of Kunming Institute of Botany, Chinese Academy of Sciences (Xiang963). Total genomic DNA was extracted to construct a library for sequencing by using Illumina sequencing methods at the Beijing Novogene Bioinformatics Technology Center. The clean data of *S. erianthum* was assembled using NOVOPlasty v3.1 (Dierckxsens et al. 2017). Assembled plastid genome annotation was conducted using Geseq in Chlorobox web service (Tillich et al. 2017), with manual corrections for start and stop codons using Geneious v.9.0.2 (Kearse et al. 2012). The annotated plastid genome sequence was submitted to GenBank (MW420931).

In this study, the chloroplast genome size is 156,343 bp, including a LSC region of 86,855 bp, a SSC region of 18,608 bp and two inverted repeats (IR). The GC content is 37.7%. This complete chloroplast genome includes a total of 128 unique genes including 83 protein-coding sequences, 37 tRNA and 8 rRNA genes.

To obtain insight explore the position of *S. erianthum* within Solanaceae, phylogenetic analyses were performed using maximum likelihood (ML) in RAxML8.0 with 1000 bootstrap replicates (Stamatakis 2014) (Figure 1) . Based on previous studies, the ML phylogenetic reconstructed based on whole chloroplast genomes from 62 Solanum plants and one sample *Eucommia ulmoides* as outgroup plants (Li et al. 2021). ML analysis showed that *S. erianthum* was the most closely related species to *S. violaceum* with 100% bootstrap values. The result is similar to the previous studies, can fully proved the classification status of *S. erianthum.* And the ML phylogenetic show *Solanum* with two clades, corresponded to the morphologically hairy and glabrous (Zhang et al. 1994). So, the complete chloroplast genome of *S. erianthum* will provide a valuable genomic basis for further studies to reconstruct its phylogeny, assess genetic variation, and develop conservation strategies when appropriate.

**Figure 1.**
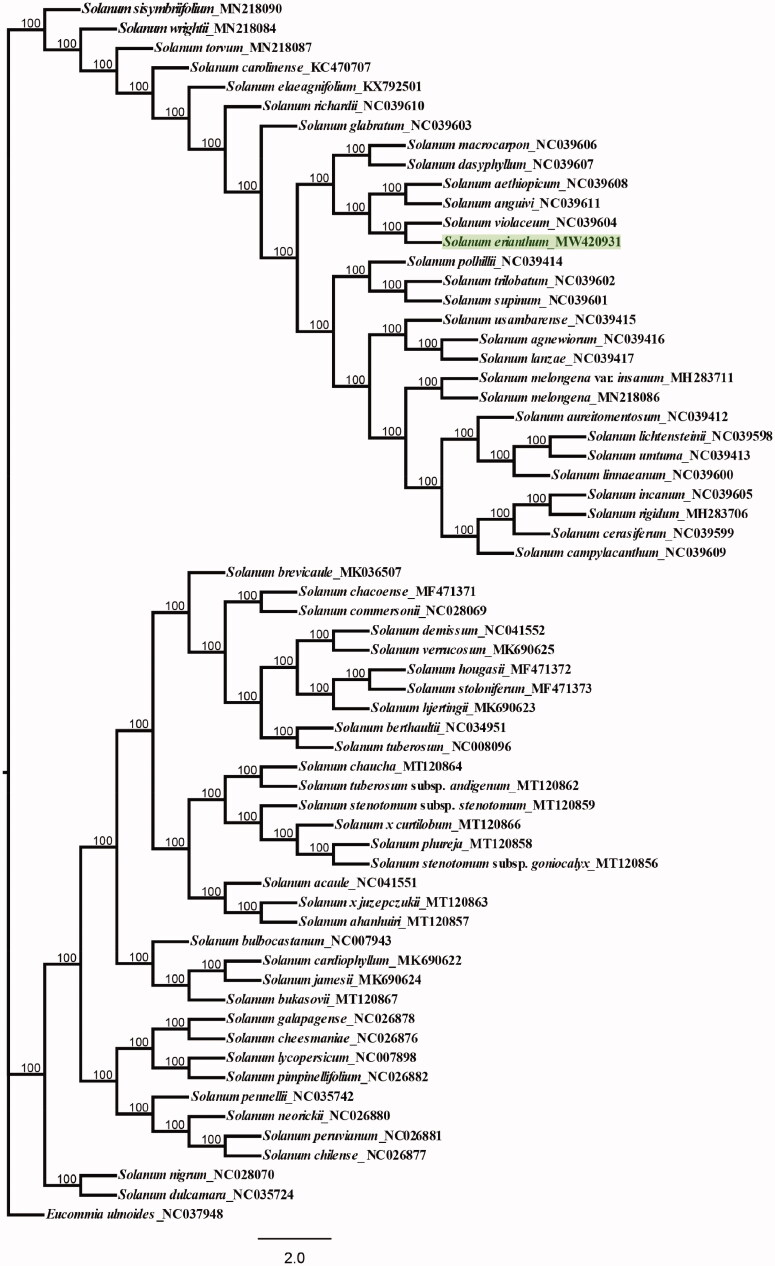
Maximum-likelihood phylogenetic tree based on the chloroplast genome sequences. Numbers at nodes represent bootstrap support values.

## Data Availability

The genome sequence data that support the findings of this study is openly available in GenBank of NCBI at https://www.ncbi.nlm.nih.gov/ under the accession no. MW420931. The associated BioProject, SRA, and Bio-Sample numbers are PRJNA689102, SRR13341520 and SAMN17199541.
